# Effect of Remimazolam and Propofol on Blood Glucose and Serum Inflammatory Markers in Patients with Type 2 Diabetes: A Clinical Trial with Prospective Randomized Control

**DOI:** 10.3390/medicina61030523

**Published:** 2025-03-17

**Authors:** Sang Hun Kim, Sang Min Yoon, Ji Hye Ahn, Yoon Ji Choi

**Affiliations:** 1 Department of Anesthesiology and Pain Medicine, College of Medicine, Chosun University, Gwangju 61453, Republic of Korea; ksh3223@chosun.ac.kr; 2 Department of Anesthesiology and Pain Medicine, Korea University Ansan Hospital, Korea University College of Medicine, Ansan 15355, Republic of Korea; ekha00041@gmail.com; 3 Department of Anesthesiology, Donghoon Advanced Lengthening Reconstruction Institute, Seongnam 13647, Republic of Korea; anji1030@naver.com

**Keywords:** anti-inflammatory markers, blood sugar, glucose, insulin, propofol, remimazolam

## Abstract

*Background and Objectives:* Patients with type 2 diabetes are at a higher risk of postoperative complications, such as infections, delayed wound healing, and increased mortality compared to non-diabetic patients. *Materials and Methods:* This prospective randomized study aims to compare the effects of two anesthetics, remimazolam and propofol, on blood glucose levels and immune function in diabetic patients undergoing surgery. Seventy-four diabetic patients undergoing general anesthesia were randomly assigned to receive either remimazolam or propofol. Plasma blood glucose levels, anti-inflammatory markers, and insulin levels were measured during the perioperative period. *Results:* No statistically significant differences were observed between the remimazolam and propofol groups in terms of neutrophil-to-lymphocyte ratio, anti-inflammatory markers, or glucose levels during the perioperative period (*p* value > 0.05). *Conclusions:* These results suggest that there is no difference between propofol and remimazolam in immune function deterioration that occurs due to surgical stress. This study is limited by its small sample size, and in future, larger trials could be conducted to find differences in the effects of blood sugar levels and serum inflammatory markers between the two groups.

## 1. Introduction

The risk of postoperative complications is higher in diabetic patients than in non-diabetic patients [1]. Diabetes-related perioperative challenges, including infection, delayed wound healing, and postoperative mortality, can arise from unexpected elevated blood sugar levels in these individuals [2]. In fact, diabetic patients have impaired immunity compared to non-diabetic patients, with elevated levels of blood glucose and cytokines such as TNF-α (tumor necrosis factor α), IL-6 (interleukin-6), and IL-8 (interleukin-8) [3]. Therefore, the increased risk of perioperative complications in diabetic patients compared to non-diabetic patients can be attributed to elevated blood glucose levels and cytokine activity.

Surgical procedures trigger physiological changes such as elevated levels of catecholamines, cortisol, and inflammatory cytokines, which in turn reduce insulin sensitivity and enhance the secretion of glucagon and growth hormone [4]. Anesthetic agents have been shown to suppress immune function in in vitro studies [5]. They have also been associated with impaired neutrophil and monocyte activity, as well as variable effects on the release of inflammatory biomarkers [5,6]. These hormonal alterations are more pronounced in patients with diabetes mellitus, leading to a catabolic state characterized by increased gluconeogenesis, glycogenolysis, lipolysis, proteolysis, and ketogenesis, which ultimately contributes to hyperglycemia [4].

Remimazolam is a short-acting benzodiazepine that acts as a GABA_A receptor agonist, inducing sedation with minimal hemodynamic instability [7]. Its potential effect on glycemic control may be related to the modulation of stress responses, as anesthesia alters cortisol and catecholamine levels, which affect glucose metabolism by influencing the hypothalamic–pituitary–adrenal (HPA) axis. Compared with other anesthetics, remimazolam induces less sympathetic activation, which may help reduce hepatic glucose output and maintain glucose homeostasis [8]. Although not primarily known for their anti-inflammatory properties, some studies have suggested that benzodiazepines may indirectly contribute to glucose stability by inhibiting the release of inflammatory cytokines [9]. However, further studies are needed to clarify the exact metabolic effects.

Therefore, this study aims to investigate whether remimazolam is superior to propofol in promoting glycemic control and enhancing inflammatory markers in diabetic patients.

## 2. Materials and Methods

This study was approved by the Institutional Review Board of Korea University Ansan Hospital (IRB number 2021AS0324) and Clinical Research information Service Number (KCT0008909, approval date: 30 October 2023). Written informed consent was obtained from all subjects who participated in this study. Seventy-four adult patients scheduled for elective spine surgery at the Korea University Ansan Hospital between November 2021 and March 2023 were enrolled consecutively. All adult type 2 diabetes patients (30–70 years old) with class I, II, or III physical status based on the American Society of Anesthesiologists (ASA) classification undergoing general anesthesia were screened for inclusion according to the study protocol. Exclusion criteria included severe systemic diseases, autoimmune diseases, and congenital disorders. All participants had to understand, express themselves, and write in Korean and give written informed consent.

Enrolled patients were randomly assigned 1:1 to the remimazolam or propofol group using computer-generated randomization. The patients were randomly allocated to receive either propofol (*n* = 36) or remimazolam (*n* = 36). For the propofol group, anesthesia was induced with 1–2 mg/kg propofol (Fresofol^®^ MCT 2%, Fresenius Kabi, Germany) and 1–2 μg/kg fentanyl. Neuromuscular blockade was achieved with 0.6–1.2 mg/kg rocuronium and the trachea was intubated. During the maintenance of anesthesia, Agilia TCI pumps were used to administer TCI propofol (at target blood concentrations of 0.5 and 6.0 μg/mLz) using the Marsh model. For the remimazolam group, anesthesia was induced with 0.2 mg/kg remimazolam (Byfavo; Hana Pharm Co., Ltd., Seoul, Republic of Korea) and 1–2 μg/kg fentanyl. Neuromuscular blockade was achieved with rocuronium, as before, and the trachea was intubated. Anesthesia was maintained with remimazolam tosylate (0.4–1.2 mg/kg/h). Remifentanil was infused throughout surgery in all patients (both the propofol and the remimazolam groups). The anesthesiologist adjusted the drug dose according to the intensity of the surgical stimulus, the patient’s vital signs, and the value of the EEG bispectral index (BIS, maintained within 40~60). Glucose-containing fluids, glucocorticoid drugs, and nonsteroidal analgesics were not allowed during the surgery.

Peripheral venous blood samples (5 mL) were collected from patients 1 day before surgery, 10 min after intubation just before skin incision, and 1 day after surgery, respectively. The levels of IL-1β (interleukin-1β), IL-6, IL-10 (interleukin-10), and TNF-α were measured in each of the blood samples using the Luminex platform (Luminex Corp., Austin, TX, USA) and serum insulin. Glucagon levels were detected by enzyme-linked immunosorbent assay (ELISA) using a Human Insulin Quantikine ELISA Kit with sensitivity of 2.15 pmol/L at the laboratory of the Woongbee Meditech. An HMX device (Beckman Coulter, Inc., Fullerton, CA, USA) was used for lymphocyte and neutrophil counts.

All patients were followed up until being discharged and were investigated for adverse effects.

IBM SPSS Statistics 26.0 software was used for data analysis. Continuous measurement data were expressed as means ± standard deviations, and statistical analyses were performed by an independent-samples t-test and a repeated-measures ANOVA (analysis of variance); count data were expressed as numbers (percentages), and statistical analyses were performed by the chi-square test and Fisher’s exact probability method. *p* < 0.05 was considered to indicate a statistically significant difference.

A quantitative approach conducted via an experimental sampling technique was employed in this research. The sample size was estimated using the G-power 3.197 software. Considering power = 0.8, α = 0.05, effect size = 0.8, and a two-tailed test, a sample size of 52 patients was calculated and recruited. The sample was randomized via the randomizer.org software. Data were analyzed using SPSS Version 21.0 (Chicago, IL, USA).

## 3. Results

**Figure 1:**
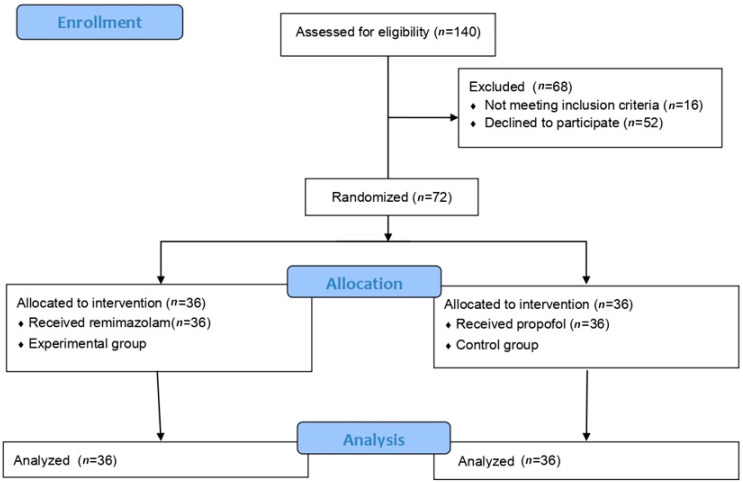
Flow diagram of this study.

Patients were categorized into the remimazolam (*n* = 36) and propofol (*n* = 36) groups based on the anesthetic agent used for induction and maintenance (Figure 1).

There were no significant differences in baseline characteristics, including age, sex, body mass index (BMI), HbA1c (glycosylated hemoglobin concentration), or underlying comorbidities, between the two groups (Table 1). Cardiovascular diseases included arrhythmias and angina that did not require treatment or were well controlled with medication and treatment. Endocrine diseases included diseases related to the thyroid gland that did not require treatment or were well controlled with medication. A glomerular filtration rate below 60 may indicate nephropathy.

**Figure 2:**
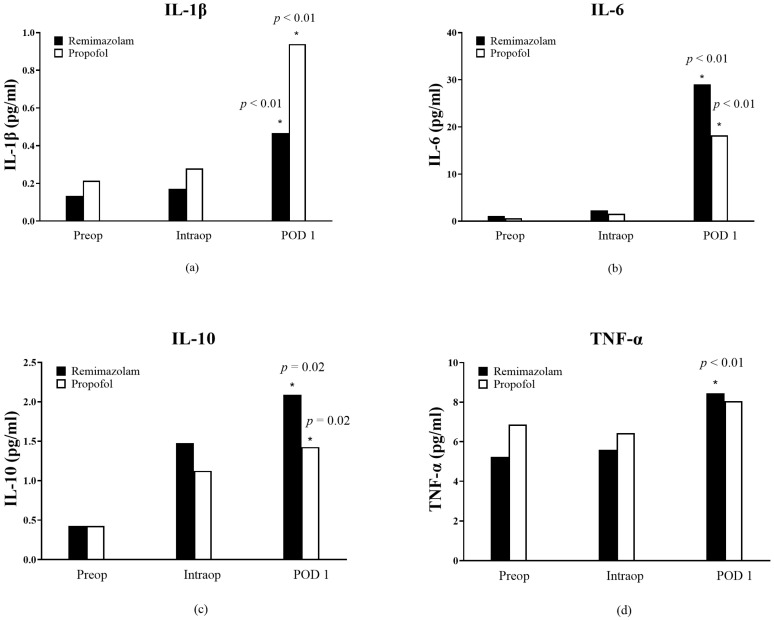
Changes in (**a**) the level of IL-1β (**b**) IL-6, (**c**) IL-10, and (**d**) TNF-α from preoperatively (preOp) to intraoperatively (intraOp) and postoperative day 1 (POD1). The asterisk * indicates *p* < 0.05 versus preop in each group.

No statistically significant differences in IL-1β, IL-6, IL-10, and TNF-α levels were observed between the remimazolam and propofol groups (Figure 2a–d).

IL-1β levels were significantly higher on POD 1 compared to preoperative levels in both groups (remimazolam group: 0.47 ± 0.51 vs. 0.13 ± 0.19, *p* < 0.01; propofol group: 0.94 ± 1.22 vs. 0.21 ± 0.10, *p* < 0.01) (Figure 2a). IL-6 and IL-10 levels were significantly higher on POD 1 compared to preoperative levels in both groups (remimazolam group: IL-6, *p* < 0.01; IL-10, *p* = 0.02; propofol group: IL-6, *p* < 0.01; IL-10, *p* = 0.02) (Figure 2b,c). TNF-α levels increased significantly on POD 1 compared to preoperative levels in the remimazolam group only (8.45 ± 7.03 vs. 5.24 ± 2.43, *p* < 0.01) (Figure 2d).

**Figure 3:**
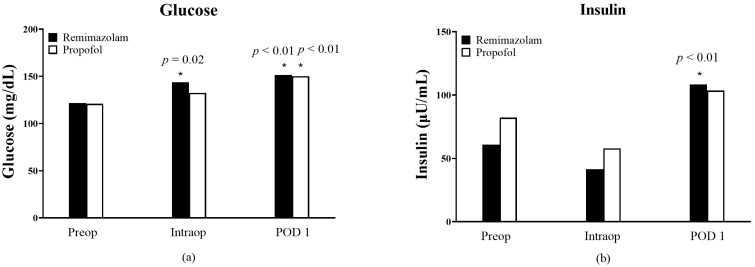
Changes in (**a**) the level of glucose and (**b**) insulin from preoperatively (preop) to intraoperatively (intraop) and postoperative days 1 (POD1). The asterisk * indicates *p* < 0.05 and versus preop in each group.

No statistically significant differences in blood glucose levels were observed between the remimazolam and propofol groups (Figure 3a). Within the remimazolam group, blood glucose levels increased significantly from the preoperative period to both the intraoperative period and POD1 (preoperative: 121.8 ± 22.0 vs. intraoperative: 143.7 ± 38.5, *p* = 0.02; POD1: 151.4 ± 35.3, *p* < 0.01). In the propofol group, blood glucose levels showed a statistically significant increase at POD1 compared to the preoperative period (150.2 ± 43.1 vs. 121.0 ± 25.9, *p* < 0.01). Insulin levels also showed no significant differences between the two groups (Figure 3b). However, in the remimazolam group, insulin levels were significantly elevated on POD 1 compared to preoperative levels (108.45 ± 73.42 vs. 60.81 ± 43.23, *p* < 0.01).

The differences in IL-1β, IL-6, IL-10, TNF-α, or insulin levels before and after surgery (POD1) were compared between the groups. There were no differences in IL-1β (*p* = 0.40), IL-6 (*p* = 0.41), IL-10 (*p* = 0.93), or TNF-α (*p* = 0.60) before and after surgery between the groups. The patients who participated in this study did not experience any adverse effects after surgery.

## 4. Discussion

Our study demonstrated that there were no significant differences in inflammatory marker levels (IL-1β, IL-6, IL-10, and TNF-α) between diabetic patients who received remimazolam or propofol during the perioperative period. Additionally, no significant differences in blood glucose elevation or insulin secretion were observed between the two anesthetics in diabetic patients.

Several studies [10,11,12] have investigated the association between inflammatory cytokines such as IL-1β, IL-6, IL-10, TNF-α, and insulin levels and postoperative complications. IL-1β and TNF-α are major inflammatory cytokines involved in the acute inflammatory response, and higher levels are associated with an increased risk of postoperative infection, systemic inflammatory response syndrome, and sepsis [10,11]. Another major inflammatory mediator, IL-6, has been widely studied as a predictor of postoperative complications, with higher levels being associated with prolonged hospital stays, organ dysfunction, and poor wound healing [10]. On the other hand, IL-10, an anti-inflammatory cytokine, plays a regulatory role in dampening excessive inflammation, but imbalanced IL-10 levels are associated with impaired immune responses and increased susceptibility to infection [11]. In addition, insulin resistance, which can be aggravated by surgical stress and inflammation, is associated with poor glycemic control, delayed wound healing, and increased risk of complications such as infection and thrombosis [12]. These results suggest that dysregulated inflammatory and metabolic responses contribute to adverse postoperative outcomes. Therefore, our study evaluated these factors, and propofol and remimazolam anesthesia had similar effects on sugar control in patients undergoing surgery.

During surgery, invasive surgical procedures and the resulting inflammatory response activate macrophages locally, which leads to the production of humoral factors [13]. These factors subsequently activate the endocrine, immune, and central nervous systems to elicit various biological responses [14,15,16]. Various cytokines induce complex signaling pathways and elicit a systemic inflammatory response, which may result in tissue damage and progression to multiple organ failure in some cases [15,17]. Previous studies demonstrated that anesthesia, invasive surgical procedures, and the use of sedatives in the intensive care unit may induce immunosuppression and lymphocyte dysfunction [18]. In addition, transient insulin resistance may also occur during surgical or nonsurgical trauma and critical illness [19]. Surgical stress induces metabolic and neuroendocrine changes, leading to poor blood glucose control and significant glucose fluctuations perioperatively, which can result in adverse outcomes, especially in diabetic patients [20,21].

There is increasing evidence that hyperglycemia negatively impacts clinical outcomes across various settings [22]. A retrospective study by McGirt et al. demonstrated that perioperative hyperglycemia during carotid endarterectomy is an independent risk factor for myocardial infarction (MI), stroke, transient ischemic attack (TIA), and death, regardless of a patient’s diabetic status [23]. Similarly, a prospective study by Ouattara et al. showed that intraoperative hyperglycemia is associated with increased postoperative morbidity, including cardiovascular, respiratory, renal, and neurological complications [24]. Acute hyperglycemia has been shown to impair monocyte activation, oxidative burst, and the phagocytic capacity of macrophages. Combined with increased protein breakdown caused by insulin resistance, these effects predispose patients to systemic and surgical site infections, delayed wound healing, and prolonged recovery [25,26].

Several studies have investigated the relationship between propofol and immune function. Propofol has been shown to exert positive effects on immune responses during the perioperative period [27]. The anti-inflammatory effects of propofol are attributed to its ability to modulate macrophage functions by suppressing migration, phagocytosis, and oxidative activity [28]. Studies comparing propofol with inhalational anesthetics have reported that propofol increased perioperative anti-inflammatory markers, although no significant differences were observed when compared to inhalational anesthetics [29]. Previous study demonstrated that remimazolam suppressed the phosphorylated p38 pathway by activating peripheral benzodiazepine receptors on hepatic macrophages at the cellular level, thereby reducing the levels of inflammatory cytokines produced [30]. Similarly, our study confirmed that the increase in anti-inflammatory marker levels induced by remimazolam was not significantly different from that of propofol. Therefore, remimazolam appears to exert positive effects on inflammatory marker levels during the perioperative period, compared to those observed with propofol.

The use of propofol has been associated with an alleviation of hyperglycemia. A study by Xinghui Xiong et al. reported that propofol reduced intraoperative hyperglycemia and insulin resistance more effectively than desflurane [31]. One possible explanation for this is that propofol inhibits the expression of the c-fos gene in the central nervous system, thereby suppressing HPA axis activity [32]. Another study comparing propofol and remimazolam found no significant differences in lactic acid and glucose levels before and after tracheal intubation in patients receiving remimazolam [33]. In our study, there was also no significant difference in blood glucose levels between the remimazolam and propofol groups, and both glucose and insulin levels increased postoperatively compared to preoperative levels. Since our study was conducted on diabetic patients, the results highlight that surgical stress exacerbates pre-existing insulin resistance, regardless of whether propofol or remimazolam is used. This finding indicates that there is a similar efficacy between remimazolam and propofol in managing blood glucose and insulin resistance during the perioperative period in diabetic patients.

Insulin resistance, hyperglycemia, and the inflammatory stress response appear to be interconnected [34]. IL-6 and TNF-α are known to influence lipid metabolism and GLUT4 expression, contributing to the development of insulin resistance [35,36,37]. Previous studies have demonstrated a similar pattern between perioperative IL-6 levels and plasma insulin levels [37]. However, this study did not observe such a pattern. This discrepancy may be explained by the fact that this study exclusively involved diabetic patients who already had exacerbated insulin resistance preoperatively, unlike participants in prior studies.

However, this study has some limitations. Unfortunately, it has a small study population, lacks a control group, and does not include follow-up monitoring for several weeks after surgery to better assess glucose control during the intervention period. If these measures had been included, the study could have provided more precise insights into the effects of anesthesia.

## 5. Conclusions

In this study, we investigated intraoperative glycemic changes in patients receiving propofol or remimazolam anesthesia. Our results showed that there were no significant differences in blood glucose levels between the two groups. There were also no significant differences in IL-1β, IL-6, IL-10, TNF-α, or insulin levels between the two groups. These results suggest that the two anesthetics have similar effects on intraoperative glucose metabolism and inflammatory responses. They may be considered for use in patients who require postoperative blood sugar control. However, further studies using larger sample sizes are needed to confirm these results.

## Figures and Tables

**Table 1:** Characteristics of patients undergoing general anesthesia.

	Propofol (*n* = 36)	Remimazolam (*n* = 36)	*p* Value
Age (years)	64.3 ± 11.6	65.7 ± 12.1	0.45
Sex (F/M)	20 (55.5)/16 (44.5)	17 (47.2)/19 (52.8)	0.64
BMI (kg/m^2^)	25.7 ± 3.8	25.4 ± 4.9	0.65
HTN	26 (72.2)	24 (66.6)	0.80
Cardiovascular disease	7 (19.4)	15 (41.6)	0.07
Pulmonary disease	8 (22.2)	12 (33.3)	0.43
Endocrine disease	10 (27.7)	3 (8.3)	0.06
Neurologic disorder	7 (19.4)	5 (13.8)	0.75
Nephropathy	6 (16.6)	8 (22.2)	0.77
HbA1c (%)	7.0 ± 1.3	6.8 ± 0.76	0.97

Data are presented as mean ± SD or numbers (percentages). BMI, body mass index; HTN, hypertension; HbA1c, glycosylated hemoglobin concentration.

## Data Availability

The data presented in this study are available on request from the corresponding author. Any requests will be reviewed against compliance with ethical, scientific, regulatory, and legal requirements. Requests to access the datasets should be directed to yjc78@korea.ac.kr.
